# Ceftolozane/tazobactam probability of target attainment and outcomes in participants with augmented renal clearance from the randomized phase 3 ASPECT-NP trial

**DOI:** 10.1186/s13054-021-03773-5

**Published:** 2021-10-02

**Authors:** Andrew F. Shorr, Christopher J. Bruno, Zufei Zhang, Erin Jensen, Wei Gao, Hwa-Ping Feng, Jennifer A. Huntington, Brian Yu, Elizabeth G. Rhee, Carisa De Anda, Sumit Basu, Marin H. Kollef

**Affiliations:** 1 Georgetown University, Washington, DC USA; 2 Merck & Co., Inc., 2000 Galloping Hill Road, Kenilworth, NJ 07033 USA; 3 Washington University School of Medicine, St. Louis, MO USA

**Keywords:** Hospital-acquired bacterial pneumonia, Multidrug resistance, *Pseudomonas aeruginosa*, Ventilator-associated bacterial pneumonia

## Abstract

**Background:**

The randomized, double-blind, phase 3 ASPECT-NP trial evaluated the efficacy of 3 g of ceftolozane/tazobactam (C/T) versus 1 g of meropenem infused every 8 h for 8 to 14 days for treatment of adults with hospital-acquired bacterial pneumonia (HABP) or ventilator-associated bacterial pneumonia (VABP). We assessed the probability of target attainment and compared efficacy outcomes from ASPECT-NP in participants with augmented renal clearance (ARC) versus those with normal renal function.

**Methods:**

Baseline renal function was categorized as normal renal function (creatinine clearance 80–130 mL/min) or ARC (creatinine clearance > 130 mL/min). Population pharmacokinetic models informed Monte Carlo simulations to assess probability of target attainment in plasma and pulmonary epithelial lining fluid. Outcomes included 28-day all-cause mortality and clinical cure and per-participant microbiologic cure rates at the test-of-cure visit.

**Results:**

A > 99% and > 80% probability of target attainment was demonstrated for ceftolozane and tazobactam, respectively, in simulated plasma and epithelial lining fluid. Within treatment arms, 28-day all-cause mortality rates in participants with normal renal function (C/T, *n* = 131; meropenem, *n* = 123) and ARC (C/T, *n* = 96; meropenem, *n* = 113) were comparable (data comparisons presented as rate; treatment difference [95% CI]) (C/T: normal renal function, 17.6%; ARC, 17.7%; 0.2 [− 9.6 to 10.6]; meropenem: normal renal function, 20.3%; ARC, 17.7%; − 2.6 [− 12.6 to 7.5]). Clinical cure rates at test-of-cure were also comparable across renal function groups within treatment arms (C/T: normal renal function, 57.3%; ARC, 59.4%; − 2.1 [− 14.8 to 10.8]; meropenem: normal renal function, 59.3%; ARC, 57.5%; 1.8 [− 10.6 to 14.2]). Per-participant microbiologic cure rates at test-of-cure were consistent across renal function groups within treatment arms (C/T: normal renal function, 72.2% [*n*/*N* = 70/97]; ARC, 71.4% [*n*/*N* = 55/77]; 0.7 [− 12.4 to 14.2]; meropenem: normal renal function, 75.0% [*n*/*N* = 66/88]; ARC, 70.0% [*n*/*N* = 49/70]; 5.0 [− 8.7 to 19.0]).

**Conclusions:**

C/T and meropenem resulted in 28-day all-cause mortality, clinical cure, and microbiologic cure rates that were comparable between participants with ARC or normal renal function. In conjunction with high probability of target attainment, these results confirm that C/T (3 g) every 8 h is appropriate in patients with HABP/VABP and ARC.

*Trial registration* ClinicalTrials.gov identifier: NCT02070757, registered February 25, 2014; EudraCT: 2012-002862-11.

## Background

The most common life-threatening nosocomial infection is pneumonia; nosocomial pneumonia can be further categorized as hospital-acquired bacterial pneumonia (HABP) or ventilator-associated bacterial pneumonia (VABP) [1–3]. HABP and VABP have been associated with mortality rates estimated as high as 50% [1].

HABP and VABP are often caused by drug-resistant pathogens, which are associated with increased mortality in this critically ill population [4–6]. A potential added complication in the treatment of critically ill patients with HABP/VABP is the high prevalence of augmented renal clearance (ARC) in this population [7, 8]. ARC, commonly defined as creatinine clearance (CrCl) > 130 mL/min, can result in suboptimal levels of renally eliminated antibacterial agents, such as β-lactams, thus resulting in worse treatment outcomes [8, 9].

Ceftolozane/tazobactam (C/T), a combination of a potent antipseudomonal cephalosporin (ceftolozane) with a β-lactamase inhibitor (tazobactam), has activity against many gram-negative pathogens, including multidrug-resistant strains, that can cause HABP/VABP [10]. C/T 3 g (ceftolozane 2 g/tazobactam 1 g) administered as a 1-h intravenous (IV) infusion every 8 h is approved for the treatment of adults with HABP/VABP [10, 11]. C/T is primarily excreted via the kidneys and requires dose modification in patients with moderate to severe renal impairment [10]. However, a population pharmacokinetic (PK) analysis based on data from other infection types found that no dose adjustment is necessary in patients with ARC and suggested that a high probability of target attainment (PTA) for C/T was achieved in this patient population [12].

With the approval of the higher C/T dose regimen of 3 g for the treatment of HABP/VABP and the high prevalence of ARC among patients receiving mechanical ventilation, it is important to understand whether a C/T dose adjustment is necessary for patients with HABP/VABP and ARC [9–11, 13]. The large, randomized, controlled, double-blind, phase 3 ASPECT-NP trial was conducted to evaluate C/T versus meropenem in participants with HABP/VABP receiving mechanical ventilation [11, 14]. Using data from ASPECT-NP, we conducted a post hoc analysis to assess PTA and compare efficacy outcomes among participants with ARC (CrCl > 130 mL/min) and those with normal renal function (CrCl 80 to 130 mL/min) to evaluate the recommended C/T dosing regimen specifically for participants with HABP/VABP and ARC.

## Methods

### Study objectives and design

The ASPECT-NP trial (ClinicalTrials.gov identifier: NCT02070757; protocol MK-7625A-008) assessed the safety and efficacy of C/T 3 g (ceftolozane 2 g/tazobactam 1 g) compared with meropenem 1 g; the full methodology has been published previously [14]. The primary objective of the present study was to examine 28-day all-cause mortality (ACM) and clinical and per-participant microbiologic cure rates at the test-of-cure (TOC) visit among participants with ARC (CrCl > 130 mL/min) and those with normal renal function (CrCl 80 to 130 mL/min).

### Analysis population

Eligibility criteria have been previously described [14]. Briefly, the study included participants aged ≥ 18 years with confirmed ventilated HABP (vHABP) or VABP at the time of randomization. Participants were excluded if only gram-positive pathogens were detected by gram stain, they developed end-stage renal disease (CrCl < 15 mL/min), required peritoneal dialysis or hemodialysis, had a urine output of < 20 mL/h over a 24-h period, or if they received > 24 h of systemic or inhaled antibacterial agents with gram-negative activity within 72 h before the first dose of study drug (participants were eligible for inclusion if they had persistent, worsening, or new nosocomial pneumonia despite ≥ 48 h of antibacterial therapy). The distribution of gram-negative pathogens in the C/T and meropenem groups was similar, including extended spectrum β-lactamase-producing Enterobacterales and multidrug-resistant *Pseudomonas aeruginosa*, and has been previously described [14]. Baseline CrCl was estimated using serum creatinine values, actual body weight, and a Cockroft–Gault formula specific for each sex [15].

### Study procedures and clinical assessments

As part of the randomization process to facilitate balanced distribution of high-risk participants between both treatment arms, participants were stratified by diagnosis (vHABP or VABP) and age (≥ 65 or < 65 years) before 1:1 assignment to receive C/T or meropenem as a 1-h IV infusion every 8 h for 8 to 14 days. A treatment duration of 14 days was recommended for participants with cultures positive for *Pseudomonas aeruginosa*. Empiric adjunctive gram-negative therapy with IV amikacin (15 mg/kg daily) or alternate aminoglycosides (per site-specific standard of care) was permitted at baseline for up to 72 h at sites with ≥ 15% local prevalence of meropenem-resistant *P. aeruginosa*. Empiric adjunctive gram-positive therapy (600 mg of linezolid every 12 h or site-specific standard-of-care alternative) was required for all participants until LRT culture results confirmed absence of *Staphylococcus aureus* or for a minimum of 8 days for participants with baseline cultures positive for *S. aureus*.

Samples for PK analyses were obtained on day 4 (or after day 4 if required) at the following times relative to 1 of the 3 daily study drug infusions: immediately before infusion (within 15 min), at the end of infusion, and 30 to 90 min, 2.5 to 3.5 h, and 5.0 to 6.0 h after study drug administration. Plasma ceftolozane and tazobactam concentrations were determined using validated high-performance liquid chromatography–tandem mass spectrometry.

### PTA analyses

PTA was assessed using Monte Carlo simulations based on existing population PK models describing plasma concentrations of ceftolozane and tazobactam in patients with HABP/VABP [16], and the simulations were aimed at dose justification for patients with HABP/VABP and varying degrees of renal impairment and augmented renal clearance by assessing the probability of PK/PD target attainment for ceftolozane and tazobactam. NONMEM (Version 7.3.0; Icon, plc, Dublin, Ireland) was used for Monte Carlo simulations. Because CrCl, as a measure of renal function, is a significant predictor of ceftolozane and tazobactam clearance in patients with HABP/VABP, a virtual patient population database stratified by various CrCl categories was generated with each group containing 1000 patients with pneumonia. This virtual population database was generated from a large virtual demographic dataset (*n* = 100,000) maintaining the same relationship between CrCl and weight from a pooled internal Merck demographic dataset from antibacterial (including patients with HABP/VABP enrolled in the ASPECT-NP study) and other infectious disease programs (*n* = 5152). The range of CrCl for the > 210 mL/min group was 210–312 mL/min.

Briefly, these population PK models were developed based on a previously established 2-compartment model with first-order elimination [17]. Plasma ceftolozane and tazobactam concentration data from 16 clinical studies, including ASPECT-NP, informed the plasma parameters of the population PK models. Pulmonary epithelial lining fluid (ELF) ceftolozane and tazobactam concentration data from 2 phase 1 studies, including one conducted in critically ill participants with pneumonia receiving mechanical ventilation, informed the ELF parameters of the population PK models; disposition of ceftolozane and tazobactam in ELF was described by a hypothetical link model with influx and elimination from the ELF compartment. Among the covariates identified in the existing population PK models in patients with HABP/VABP, CrCl was a significant covariate on ceftolozane and tazobactam clearance; weight and pneumonia were covariates on ceftolozane and tazobactam volume of distributions; and pneumonia was a covariate on the influx and elimination rate constants for the ELF compartment [16].

For the Monte Carlo simulations, we created a simulated population of patients with characteristics similar to that of participants from previous infectious disease clinical trials [16]. From this large pool of simulated patients, 1000 patients with paired body weight and CrCl were randomly drawn for each of the following renal function categories to estimate PTA in each of the CrCl ranges: CrCl 80 to 130 mL/min (normal renal function); CrCl > 130 to < 180 mL/min (ARC); CrCl 180 to < 210 mL/min (ARC); and CrCl ≥ 210 mL/min (ARC). The PTA was assessed based on previously established PK/pharmacodynamic (PD) targets and applied to both plasma and ELF [18–21]. The ceftolozane PK/PD target used was 50% of the dosing period that the free ceftolozane drug concentration exceeded the minimum inhibitory concentration (50% *f*T > MIC) of 4 μg/mL; this target was shown to result in a 2-log kill in a murine infection model [19]. The tazobactam PK/PD target used was 35% of the dosing period that the free tazobactam drug concentration remained above the threshold concentration (C_T_) of 1 μg/mL (35% *f*T > C_T_), restoring ceftolozane antibacterial activity to a 1-log kill [19, 20]. Free drug concentrations were computed assuming protein binding levels of 21% and 0% in plasma and ELF, respectively for ceftolozane, and 30% and 0% in plasma and ELF, respectively, for tazobactam across the range of drug concentrations [22].

### Clinical outcomes

The primary and key secondary endpoints of ASPECT-NP were 28-day ACM and clinical response at the TOC visit (7 to 14 days after the end-of-treatment visit), respectively, in the intention-to-treat (ITT) population [14]. A clinical response outcome was defined as “cure” if a surviving participant exhibited all of the following: complete resolution of all or most clinical signs and symptoms of pneumonia; no new signs, symptoms, or complications attributable to vHABP/VABP; and no receipt of additional antibacterial therapy administration for vHABP/VABP other than approved adjunctive therapy. “Clinical failure” was defined as progression, relapse, or recurrence of new symptoms; persistence or insufficient improvement of vHABP/VABP; discontinuation of study drug due to resistant LRT pathogens; need for alternative or prolonged antibacterial therapy for vHABP/VABP; or death due to vHABP/VABP. A microbiologic response defined as “cure” or “presumed cure” included eradication/presumed eradication of all baseline pathogens (≥ 1-log reduction in bacterial burden and per-pathogen count of ≤ 10^4^ colony-forming units [CFU]/mL for endotracheal aspirate or sputum specimens, ≤ 10^3^ CFU/mL for bronchoalveolar lavage specimens, and ≤ 10^2^ CFU/mL for protected brush specimens from a follow-up LRT culture) or absence of an LRT culture resulting in a patient deemed a clinical cure. Post hoc analyses were conducted to assess 28-day ACM and clinical and microbiologic cure rates at the TOC visit for participants with ARC versus those with normal renal function.

### Statistical analysis

Assessments were evaluated in the following analysis populations: ITT (all randomized participants, regardless of receipt of study drug); microbiologic ITT (mITT; participants who received any study drug and had ≥ 1 confirmed LRT pathogen that was susceptible to ≥ 1 study drug); and clinically evaluable (CE; participants who received study drug, adhered to the protocol through the TOC visit, and had an evaluable clinical outcome at the TOC visit).

This post hoc subgroup analysis was not powered to test for inferiority. For 28-day ACM, the treatment difference between renal functional groups was calculated as normal renal function minus ARC; for clinical and microbiologic response, the treatment difference was calculated as ARC minus normal renal function. The 2-sided 95% CIs for the treatment difference in proportions between ARC and normal renal function were calculated as unstratified Newcombe CIs. All statistical analyses were performed using SAS versions 9.3 and 9.4 (Cary, NC, USA).

## Results

### PK analysis

Steady-state plasma PK parameters for ASPECT-NP participants with varying renal function are summarized in Table 1. As anticipated, ceftolozane and tazobactam exposures decreased with increasing CrCl. Steady-state ELF PK parameters with varying renal function and ARC are summarized in Table 2. Similar to the results in plasma, ELF exposures for ceftolozane and tazobactam decreased with increasing CrCl.

**Table 1:** Summary of ceftolozane and tazobactam steady-state plasma exposures in patients with HABP/VABP

Exposure measure	Statistic	Ceftolozane				Tazobactam			
		CrCl ≥ 80 to < 150 mL/min	CrCL ≥ 150 to < 180 mL/min	CrCL ≥ 180 to < 210 mL/min	CrCL ≥ 210 mL/min	CrCl ≥ 80 to < 150 mL/min	CrCL ≥ 150 to < 180 mL/min	CrCL ≥ 180 to < 210 mL/min	CrCL ≥ 210 mL/min
AUC_0–8_ (μg × h/mL)	*n*	138	24	16	20	138	24	16	20
	Geometric mean (geometric %CV)	371 (55.3)	276 (52.6)	248 (59.7)	201 (67.2)	61.9 (80.1)	42.1 (56.2)	42.0 (90.7)	34.8 (85.6)
*C*_max_ (μg/mL)	*n*	138	24	16	20	138	24	16	20
	Geometric mean (geometric %CV)	103 (42.6)	88.6 (40.2)	78.0 (52.7)	65.3 (63.0)	25.8 (45.0)	21.0 (34.3)	19.6 (43.6)	18.6 (48.8)

Among all participants from the intention-to-treat population who had pharmacokinetic data collected

AUC_0–8_, area under the concentration–time curve from time 0 to 8 h after start of infusion; *C*_max_, maximum drug concentration; CrCl, creatinine clearance; CV, coefficient of variation; HABP/VABP, hospital-acquired bacterial pneumonia/ventilator-associated bacterial pneumonia

**Table 2:** Summary of ceftolozane and tazobactam steady-state pulmonary ELF exposures in patients with HABP/VABP

Exposure measure	Statistic	Ceftolozane				Tazobactam			
		CrCl ≥ 80 to < 150 mL/min	CrCL ≥ 150 to < 180 mL/min	CrCL ≥ 180 to < 210 mL/min	CrCL ≥ 210 mL/min	CrCl ≥ 80 to < 150 mL/min	CrCL ≥ 150 to < 180 mL/min	CrCL ≥ 180 to < 210 mL/min	CrCL ≥ 210 mL/min
AUC_0–8_ (μg × h/mL)	*n*	138	24	16	20	138	24	16	20
	Geometric mean (geometric %CV)	199 (78.9)	149 (64.0)	127 (86.6)	118 (69.6)	24.7 (108)	14.4 (72.1)	16.0 (85.9)	13.6 (77.5)
*C*_max_ (μg/mL)	*n*	138	24	16	20	138	24	16	20
	Geometric mean (geometric %CV)	28.3 (78.8)	21.0 (69.8)	17.4 (87.8)	15.9 (71.7)	4.68 (107)	2.78 (65.7)	3.31 (92.9)	2.81 (85.6)

Among all participants from the intention-to-treat population who had pharmacokinetic data collected

AUC_0–8_, area under the concentration–time curve from time 0 to 8 h after start of infusion; *C*_max_, maximum drug concentration; CrCl, creatinine clearance; CV, coefficient of variation; ELF, epithelial lining fluid; HABP/VABP, hospital-acquired bacterial pneumonia/ventilator-associated bacterial pneumonia

### PTA analysis

**Fig. 1:**
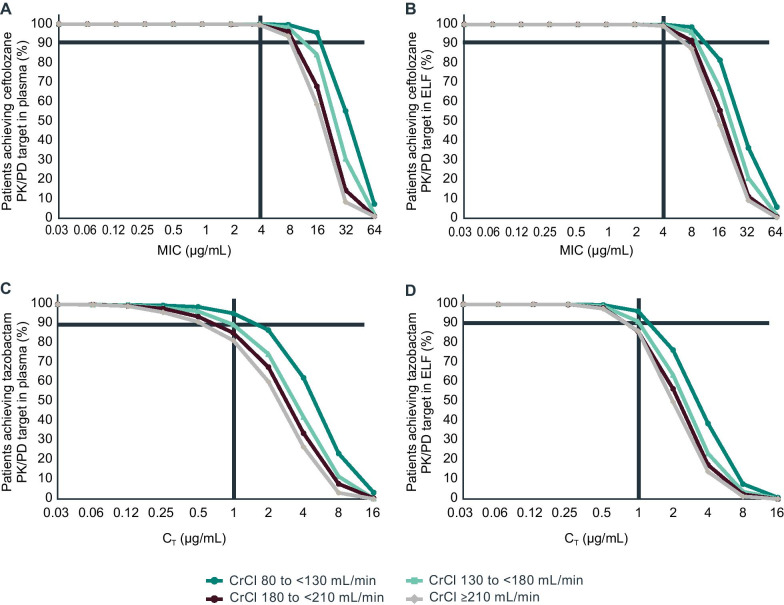
PTA for ceftolozane and tazobactam in simulated patients with vHABP/VABP by renal function. Panels A and B represent PTA for ceftolozane at a target of 50% *f*T > MIC. Panels C and D represent PTA for tazobactam at a target of 35% *f*T > C_T._ Left panels represent PTA in plasma and right panels represent PTA in ELF. Solid horizontal line on plots represents 90% PTA; vertical line in panels A and B represents an MIC of 4 μg/mL; vertical line in panels C and D represents a *C*_T_ of 1 μg/mL. *CrCl* creatinine clearance, *C*_T_ threshold concentration, *ELF* epithelial lining fluid, *fT* > *C*_T_ time period that the free drug concentration remained above the threshold concentration, *fT* > *MIC* time period that the free drug concentration exceeded the MIC value, *MIC* minimum inhibitory concentration, *PD* pharmacodynamic, *PK* pharmacokinetic, *PTA* probability of target attainment, *vHABP/VABP* ventilated hospital-acquired bacterial pneumonia/ventilator-associated bacterial pneumonia

Monte Carlo simulation results demonstrated high PTA in plasma and ELF for both ceftolozane and tazobactam at the C/T 3-g dose (ceftolozane 2 g/tazobactam 1 g) approved for treatment of HABP/VABP. More than 99% of all simulated patients were estimated to achieve the ceftolozane PK/PD target in both plasma and ELF across all renal function groups, with no notable differences between plasma and ELF or across patients of different ARC categories (Fig. 1A, B). The tazobactam PK/PD target was achieved by > 80% of simulated patients in both plasma and ELF. The PTA for tazobactam in ELF was slightly higher than that of plasma for simulated patients with CrCl of ≥ 210 mL/min but was roughly equivalent between ELF and plasma for all other renal function categories (Fig. 1C, D). While high PTA for tazobactam was estimated for all simulated patients regardless of renal function, an incremental decrease in PTA was observed with increased ARC across the 3 simulated ARC categories.

### Clinical outcomes

Among the 726 participants randomized in ASPECT-NP, 254 (35%) had normal renal function (CrCl 80 to 130 mL/min) and 209 (29%) had ARC (CrCl > 130 mL/min). Within both treatment arms, the proportion of participants with ARC at baseline ranged from 27 to 31% across the various analysis populations. Demographics and baseline characteristics are summarized in Table 3. Compared with the normal renal function groups, the ARC groups in both treatment arms had younger participants and a greater proportion of males. Within the C/T arm, Sequential Organ Failure Assessment (SOFA) scores, Acute Physiology and Chronic Health Evaluation II (APACHE II) scores, Clinical Pulmonary Infection Scores (CPIS), rates of vHABP diagnosis, and previous antibacterial agent use were comparable between the normal renal function group and the ARC group; however, the rate of bacteremia was higher and duration of hospitalization was longer in the ARC group. Within the meropenem treatment arm, participants with ARC had lower SOFA and APACHE II scores compared with participants with normal renal function. Other baseline demographics and clinical characteristics were generally well balanced between normal renal function and ARC groups within each treatment arm.

**Table 3:** Baseline patient demographics and clinical characteristics in the ITT population by renal function group

ITT population	Ceftolozane/tazobactam		Meropenem	
	Normal renal function (*n* = 131)	Augmented renal clearance (*n* = 96)	Normal renal function (*n* = 123)	Augmented renal clearance (*n* = 113)
Male	93 (71.0)	78 (81.3)	84 (68.3)	89 (78.8)
Age (years)				
< 65	75 (57.3)	78 (81.3)	74 (60.2)	96 (85.0)
≥ 65	56 (42.7)	18 (18.8)	49 (39.8)	17 (15.0)
Mean (SD)	59.6 (16.5)	49.3 (15.1)	58.7 (15.6)	48.2 (15.6)
Median (IQR)	63.0 (49.0–71.0)	50.0 (40.5–60.0)	61.0 (50.0–71.0)	49.0 (36.0–60.0)
Weight, kg, median (IQR)	80.0 (72.0–90.0)	85.0 (75.0–95.5)	79.3 (70.0–90.0)	80.0 (72.0–95.0)
Body mass index, kg/m^2^, median (IQR)	26.8 (24.1–29.4)	27.8 (25.1–31.1)	26.3 (24.0–29.4)	27.1 (24.2–30.9)
Baseline CrCl, mL/min, median (IQR)	99.0 (90.0–112.0)	172.3 (145.3–204.4)	100.5 (90.0–113.5)	164.0 (139.6–204.0)
Randomized while in ICU	122 (93.1)	89 (92.7)	113 (91.9)	109 (96.5)
APACHE II score				
< 10	7 (5.3)	8 (8.3)	11 (8.9)	14 (12.4)
10–14	25 (19.1)	17 (17.7)	19 (15.4)	25 (22.1)
15–19	61 (46.6)	45 (46.9)	53 (43.1)	49 (43.4)
20–24	27 (20.6)	20 (20.8)	27 (22.0)	20 (17.7)
25–35	11 (8.4)	6 (6.3)	13 (10.6)	4 (3.5)
≥ 35	0	0	0	0
Missing	0	0	0	1 (0.9)
Mean (SD)	17.2 (4.8)	16.5 (4.9)	17.4 (5.5)	15.6 (5.1)
Median (IQR)	18.0 (15.0–20.0)	16.0 (14.0–20.0)	17.0 (15.0–21.0)	16.0 (12.5–19.0)
Previous antibacterial agent use^a^				
Yes	114 (87.0)	88 (91.7)	111 (90.2)	100 (88.5)
No	17 (13.0)	8 (8.3)	12 (9.8)	13 (11.5)
Primary diagnosis				
vHABP	26 (19.8)	18 (18.8)	33 (26.8)	15 (13.3)
VABP	105 (80.2)	78 (81.3)	90 (73.2)	98 (86.7)
SOFA score				
≤ 7	108 (82.4)	77 (80.2)	82 (66.7)	80 (70.8)
> 7	23 (17.6)	19 (19.8)	41 (33.3)	33 (29.2)
PaO_2_/FiO_2_ ratio (mmHg)				
≤ 240	100 (76.3)	61 (63.5)	92 (74.8)	83 (73.5)
> 240	30 (22.9)	33 (34.4)	31 (25.2)	29 (25.7)
Clinical Pulmonary Infection Score (CPIS)				
Missing	1 (0.8)	2 (2.1)	0	1 (0.9)
< 6	9 (6.9)	6 (6.3)	7 (5.7)	14 (12.4)
7	11 (8.4)	5 (5.2)	11 (8.9)	17 (15.0)
8	19 (14.5)	13 (13.5)	19 (15.4)	11 (9.7)
> 8	92 (70.2)	72 (75.0)	86 (69.9)	71 (62.8)
Duration of hospitalization (days)^b^				
< 5	21 (16.0)	23 (24.0)	30 (24.4)	25 (22.1)
≥ 5	110 (84.0)	71 (74.0)	92 (74.8)	88 (77.9)
Missing	0	2 (2.1)	1 (0.8)	0
Mean (SD)	10.6 (7.8)	9.0 (6.5)	9.6 (7.2)	8.8 (6.4)
Median (IQR)	8.0 (6.0–13.0)	8.0 (5.0–12.0)	7.5 (5.0–12.0)	7.0 (5.0–11.0)
Duration of mechanical ventilation (days)^b^				
< 5	54 (41.2)	40 (41.7)	59 (48.0)	51 (45.1)
≥ 5^c^	77 (58.8)	55 (57.3)	64 (52.0)	61 (54.0)
Missing	0	1 (1.0)	0	1 (0.9)
Mean (SD)	7.2 (6.3)	21.5 (99.6)	7.0 (6.1)	7.5 (7.4)
Median (IQR)	5.6 (3.5–9.2)	5.9 (3.5–10.5)	5.5 (2.8–9.0)	5.5 (3.5–9.2)
Previous unsuccessful antibacterial therapy for current episode of vHABP/VABP^d^				
Yes	21 (16.0)	14 (14.6)	12 (9.8)	14 (12.4)
No	110 (84.0)	82 (85.4)	111 (90.2)	99 (87.6)
Bacteremia (gram-negative respiratory pathogen)				
Yes	4 (3.1)	7 (7.3)	4 (3.3)	10 (8.8)
No	127 (96.9)	89 (92.7)	119 (96.7)	103 (91.2)

Data are shown as *n* (%) unless otherwise specified

*APACHE II* Acute Physiology and Chronic Health Evaluation II, *CrCl* creatinine clearance, *ICU* intensive care unit, *IQR* interquartile range, *ITT* intention-to-treat, *SOFA* Sequential Organ Failure Assessment, *VABP* ventilator-associated bacterial pneumonia, *vHABP* ventilated hospital-acquired bacterial pneumonia

^a^Antibacterial therapy received in the 14 days before the first dose of study drug

^b^Before randomization

^c^Some of these patients may have been unsuccessfully treated with antibacterial therapy for the current episode of vHABP/VABP before randomization, and the denominator includes patients with vHABP; thus, this number is not an exact substitute for late VABP

^d^Persistent or worsening signs/symptoms of vHABP/VABP after ≥ 48 h of antibacterial therapy against gram-negative pathogens

**Fig. 2:**
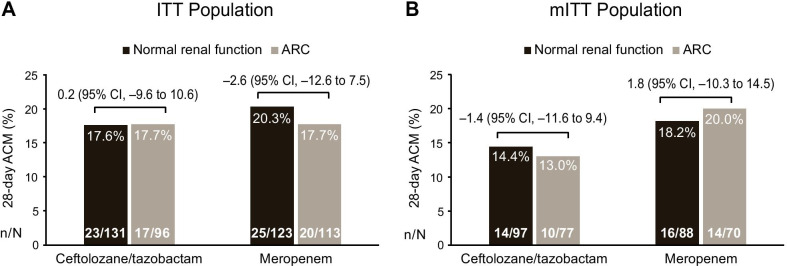
28-day ACM in ceftolozane/tazobactam- or meropenem-treated patients by renal function. *ACM* all-cause mortality*, ARC* augmented renal clearance, *ITT* intention-to-treat, *mITT* microbiologic intention-to-treat

In the ITT and mITT populations, 28-day ACM rates were comparable between participants with normal renal function and ARC in the C/T arm (ITT: normal renal function, 17.6%; ARC, 17.7%; treatment difference, 0.2 [95% CI, − 9.6 to 10.6]; mITT: normal renal function, 14.4%; ARC, 13.0%; treatment difference, − 1.4 [95% CI, − 11.6 to 9.4]; Fig. 2). In the meropenem treatment arm, the 28-day ACM rates for participants in the normal renal function and ARC groups were also comparable in both the ITT and mITT populations (Fig. 2).

**Fig. 3:**
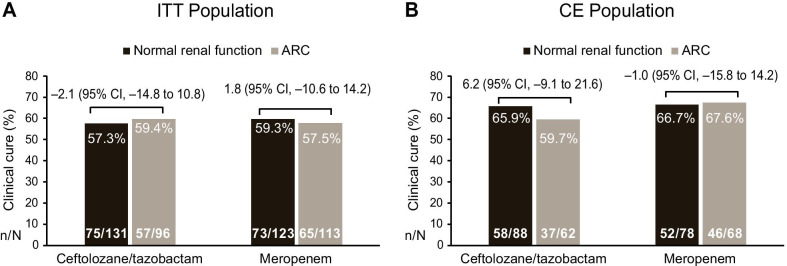
Clinical cure at the test-of-cure visit in ceftolozane/tazobactam- or meropenem-treated patients by renal function. *ARC* augmented renal clearance, *CE* clinically evaluable, *ITT* intention-to-treat

In the C/T treatment arm, more than half of all participants in the ITT and CE populations achieved clinical cure at the TOC visit regardless of renal function status (ITT: normal renal function, 57.3%; ARC, 59.4%; treatment difference, − 2.1 [95% CI, − 14.8 to 10.8]; CE: normal renal function, 65.9%; ARC, 59.7%; treatment difference, 6.2 [95% CI, − 9.1 to 21.6]; Fig. 3). Clinical cure rates in the meropenem treatment arm were similar between the normal renal function and ARC groups in both the ITT and CE populations (Fig. 3).

**Fig. 4:**
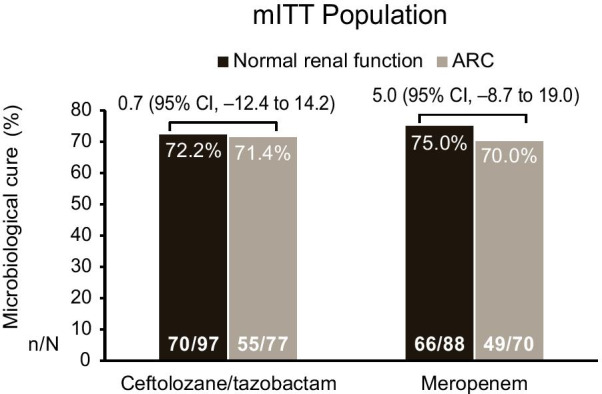
Per-patient microbiologic cure at the test-of-cure visit in ceftolozane/tazobactam- or meropenem-treated patients by renal function. *ARC* augmented renal clearance, *mITT* microbiologic intention-to-treat

The per-participant microbiologic cure rates at the TOC visit in the mITT population were also comparable across renal function groups in the C/T and meropenem treatment arms (C/T: normal renal function, 72.2%; ARC, 71.4%; treatment difference, 0.7 [95% CI, − 12.4 to 14.2]; meropenem: normal renal function, 75.0%; ARC, 70.0%; treatment difference, 5.0 [95% CI, − 8.7 to 19.0]; Fig. 4).

## Discussion

The PTA analysis results presented herein support previous findings that demonstrate high PTA with C/T 3 g dosed every 8 h in patients with HABP/VABP and in critically ill participants with and without ARC, without a need for further dose modification [22, 23]. The renal clearance of antibacterial therapeutics has a direct impact on PK. In the case of critically ill patients with ARC, enhanced drug clearance may lead to lower maximum plasma concentration, shorter drug half-life, and lower area under the concentration–time curve, potentially contributing to therapeutic failure [8]. This point is particularly important for drugs such as ceftolozane and tazobactam, which are both renally eliminated and for which efficacy is time-dependent and relies on the *f*T > MIC and *f*T > *C*_T_, respectively [8, 10, 11]. Previous PTA analyses conducted before the ASPECT-NP study demonstrated high ceftolozane and tazobactam PTA in plasma and ELF [12, 22]. The current PK analysis expands on the previously published C/T population PK models in two important ways [12, 22]. First, we honed the existing model through inclusion of clinical data from the ASPECT-NP trial to incorporate the effect of pneumonia and ARC on the PK of ceftolozane and tazobactam. In addition, we included data from a phase 1 study that collected ELF PK from critically ill participants with confirmed or suspected pneumonia to provide additional data in the relevant population to increase confidence in the reliability of simulations from previous population PK analyses [18].

The simulated PTA analyses employed in this study used stratified ARC categories: CrCl > 130 to < 180 mL/min, 180 to < 210 mL/min, and ≥ 210 mL/min. With this approach, a high PTA for ceftolozane and tazobactam was observed in both plasma and ELF for all renal function categories, including those with the greatest ARC severity.

ARC occurs in a substantial proportion of critically ill patients including those with HABP/VABP [7–9, 13]. In line with these observations, the ASPECT-NP trial enrolled a significant proportion of participants with ARC. To better understand the impact of ARC on treatment with C/T or meropenem, this post hoc analysis directly compared efficacy results among participants from ASPECT-NP with normal renal function versus those with ARC. Overall, 28-day ACM, clinical cure, and microbiologic cure rates were comparable between normal renal function and ARC groups in both treatment arms. Overall, rates of 28-day ACM and clinical cure in both treatment arms were comparable with rates reported for an analysis of 4 previous phase 3, multinational, antibacterial clinical trials in participants with HABP/VABP caused by gram-negative pathogens (pooled 28-day ACM, 17.1%; pooled clinical success rate, 59.9%) [24].

In the C/T treatment arm, participants with ARC had comparable CPIS, SOFA scores, and APACHE II scores and similar rates of previous antibacterial agent use and days of ventilation before randomization, yet higher rates of bacteremia and longer duration of hospitalization. Overall, the demographic and baseline data in the C/T arm show that the ARC group was younger and predominantly male, similar to other studies of ARC, but did not differ substantially from participants with normal renal function in terms of HABP/VABP severity [8, 9, 25–28].

The previously published results from the overall population of the ASPECT-NP trial demonstrated that C/T was noninferior to meropenem for both the primary endpoint of 28-day ACM and the key secondary endpoint of clinical response at the TOC visit [14]. The results of the ASPECT-NP study contrast with other recent phase 3 HABP/VABP studies of novel antibacterial agents that failed to show noninferiority [26, 29, 30]. The failure of doripenem to show noninferiority may be on account of the difference in treatment regimen durations between comparator arms, with a 7-day doripenem regimen versus a 10-day imipenem-cilastatin regimen [31]. In the tigecycline and ceftobiprole studies, the experimental therapy was given at standard doses that had previously shown to be efficacious for non-HABP/VABP indications [29, 30, 32, 33]. ARC has been associated with subtherapeutic concentrations of β-lactam antibacterials and higher rates of antibacterial treatment failure, suggesting a need for modified dosing regimens to maintain efficacy in these critically ill patients [34, 35]. Thus, the presence of ARC in the critically ill HABP/VABP study population of the tigecycline and ceftobiprole trials may have led to underdosing and contributed to treatment failure [36]. Based on prior population PK modeling that balanced the effect of ARC and the need to achieve therapeutic concentrations in ELF, a C/T dosing regimen of 3 g every 8 h (which is double the dose approved for treatment of complicated urinary tract infection and complicated intra-abdominal infection) was chosen for study in the ASPECT-NP trial. In a previous phase 1 study of critically ill participants with ARC, confirmed by direct CrCl measurement from urine, the 3 g C/T dose achieved PK/PD targets and was determined to be appropriate for patients with ARC [37]. In the current analysis, the comparable 28-day ACM, clinical cure, and microbiologic cure rates between participants with normal renal function and those with ARC underscore the appropriateness of the C/T dosing regimen chosen for this study. Results held across all study populations including the ITT, CE, and mITT, highlighting the robustness of these findings.

The comparator, meropenem, used in the ASPECT-NP study was dosed at 1 g every 8 h, which is the recommended dose in both the meropenem label and in the international treatment guidelines for HABP/VABP [1]. A PK modeling study suggested that a higher dose of meropenem (2 g every 8 h) or dosing by extended infusion may be required for treatment of HABP/VABP [38]. As meropenem PK sampling was not conducted in the ASPECT-NP study, it is not possible to comment on the relationship between meropenem exposure and response. However, if the meropenem dosing used in this study resulted in clinically relevant underexposure, it would be expected to manifest as lower efficacy and be most evident in participants with ARC. Meropenem-treated participants had 28-day ACM rates as well as clinical and microbiologic response rates that were comparable between the normal renal function and ARC groups, suggesting that the meropenem dosing used in this trial was adequate for participants with ARC. However, the meropenem dosing regimen used in this trial may not be appropriate for all patients with HABP/VABP.

The current analysis is not without limitations. The categorization of ARC was based on baseline-assessed CrCl measurement without postbaseline reclassification of renal function category or assessment of ARC duration. Specifically, for this analysis, participants who entered the study with ARC but later reverted to normal renal function or developed renal insufficiency would have had their therapy dosing adjusted based on their changing renal function but would have remained categorized as having ARC. However, published data suggest that the presence of ARC upon admission to the intensive care unit strongly predicts sustained CrCl elevation at 1 week, suggesting that ARC would have persisted throughout at least the first 7 days of study therapy in most participants [9]. Additionally, use of the Cockroft–Gault formula to estimate baseline CrCl in lieu of direct measurement from urine samples is also a limitation. In patients with obesity, use of the Cockroft–Gault formula may result in overestimation of CrCl [39]. Although this study population included a substantial number of participants with ARC, the absolute size of this subgroup was still relatively small; thus, the analysis was underpowered to detect statistical differences in clinical outcomes between the C/T-treated participants with ARC and meropenem-treated participants with ARC. Therefore, this analysis focused on differences between renal subgroups within each treatment arm but was not powered for hypothesis testing of such comparisons; it is possible that differences in efficacy rates between participants with normal renal function and ARC may have been more pronounced in a larger study. Finally, the PTA analysis was conducted with simulated patients, and PK/PD data for plasma and ELF may not be representative of clinical samples.

## Conclusions

Using a PK/PD target of 50% *f*T > MIC of 4 μg/mL, the PTA for ceftolozane in plasma and pulmonary ELF was > 99% in patients with ARC. The PTA was > 80% for tazobactam in both plasma and ELF using a PK/PD target of 35% *f*T > *C*_T_ of 1 μg/mL, regardless of renal function status. Comparable 28-day ACM rates and clinical cure and microbiologic cure rates at the TOC visit were found across the C/T and meropenem treatment arms for participants with normal renal function and those with ARC. These results indicate that C/T 3 g administered every 8 h is an appropriate dose for critically ill patients with HABP/VABP and ARC.

## Data Availability

MSD’s data sharing policy, including restrictions, is available at http://engagezone.msd.com/ds_documentation.php. Requests for access to the clinical study data can be submitted through the EngageZone site or via email to dataaccess@merck.com.

## Abbreviations

ACM: All-cause mortality
APACHE II: Acute Physiology and Chronic Health Evaluation II
ARC: Augmented renal clearance
CE: Clinically evaluable
CFU: Colony-forming units
CPIS: Clinical Pulmonary Infection Score
CrCl: Creatinine clearance
C/T: Ceftolozane/tazobactam
C_T_: Threshold concentration
ELF: Epithelial lining fluid
*f*T > *C*_T_: Time period that the free drug concentration remained above the threshold concentration
*f*T > MIC: Time period that the free drug concentration exceeded the MIC value
HABP: Hospital-acquired bacterial pneumonia
ICU: Intensive care unit
IQR: Interquartile range
ITT: Intention-to-treat
LRT: Lower respiratory tract
MIC: Minimum inhibitory concentration
mITT: Microbiologic intention-to-treat
PK: Pharmacokinetic
PTA: Probability of target attainment
SOFA: Sequential Organ Failure Assessment
TOC: Test of cure
VABP: Ventilator-associated bacterial pneumonia
vHABP: Ventilated hospital-acquired bacterial pneumonia
